# The Identification of Microdeletion and Reciprocal Microduplication in 22q11.2 Using High-Resolution CMA Technology

**DOI:** 10.1155/2016/7415438

**Published:** 2016-03-31

**Authors:** Ana Julia Cunha Leite, Irene Plaza Pinto, Damiana Mirian da Cruz e Cunha, Cristiano Luiz Ribeiro, Claudio Carlos da Silva, Aparecido Divino da Cruz, Lysa Bernardes Minasi

**Affiliations:** ^1^Programa de Pós-Graduação (Mestrado) em Genética, Pontifícia Universidade Católica de Goiás, Rua 235, No. 40, Setor Leste Universitário, 74605-050 Goiânia, GO, Brazil; ^2^Núcleo de Pesquisas Replicon, Departamento de Biologia, Pontifícia Universidade Católica de Goiás, Rua 235, No. 40, Setor Leste Universitário, 74605-050 Goiânia, GO, Brazil; ^3^Programa de Pós-Graduação em Biotecnologia e Biodiversidade, Universidade Federal de Goiás, Goiânia, GO, Brazil; ^4^Laboratório de Citogenética Humana e Genética Molecular, Secretaria do Estado da Saúde de Goiás, Goiânia, GO, Brazil

## Abstract

The chromosome 22q11.2 region has long been implicated in genomic diseases. Some genomic regions exhibit numerous low copy repeats with high identity in which they provide increased genomic instability and mediate deletions and duplications in many disorders. DiGeorge Syndrome is the most common deletion syndrome and reciprocal duplications could be occurring in half of the frequency of microdeletions. We described five patients with phenotypic variability that carries deletions or reciprocal duplications at 22q11.2 detected by Chromosomal Microarray Analysis. The CytoScan HD technology was used to detect changes in the genome copy number variation of patients who had clinical indication to global developmental delay and a normal karyotype. We observed in our study three microdeletions and two microduplications in 22q11.2 region with variable intervals containing known genes and unstudied transcripts as well as the LCRs that are often flanking and within this genomic rearrangement. The identification of these variants is of particular interest because it may provide insight into genes or genomic regions that are crucial for specific phenotypic manifestations and are useful to assist in the quest for understanding the mechanisms subjacent to genomic deletions and duplications.

## 1. Introduction 

The copy number variations (CNVs) changes result in meiotic nonallelic homologous recombination (NAHR) between low copy repeats (LCR) that are often flanking these genomics rearrangements. The crossover mediated by NAHR can be interchromosomal or intrachromosomal [1]. Some genomic regions exhibit numerous LCRs with high identity sequences (95–99%) which provide increased genomic instability and mediate deletions and duplications in many disorders [2]. The microarray technology has increased the detection of CNVs and the diagnosis of patients with multiple congenital anomalies and intellectual disability.

The chromosome 22q11.2 region contains eight LCRs, designated from A to H. Four centromeric LCR22s (A to D) were related to reciprocal microduplications and DiGeorge/Velocardiofacial Syndromes (DGS/VCFS) (OMIM 188400 and 192430). On the other hand, telomeric LCR22s, named LCR22D–H, located in a distal portion of 22q11.2 region, were related to distal microdeletions and reciprocal microduplications [3, 4].

DGS is the most common deletion syndrome with an incidence of 1 : 4000 newborns and has a spectrum of clinical abnormalities that affects multiple systems, including cardiovascular, neurological, psychiatric, endocrine, and immune systems. Palatal abnormalities and characteristic facial features also can be present [5]. Around 85–90% of individuals with DGS have the deletions of 3 Mb spanning LCR22A–D, while 8–10% have a 1.5 Mb deletion in LCR22A-B [6].

Most of the individuals with 22q11.2 microduplications carry approximately 3 Mb in length, among LCR22A and LCR22D, which are the reciprocal of the common deleted region found in DGS/VCFS, while few patients have 1.5 Mb duplications among LCR22A and LCR22B [7]. Larger duplications of 4 Mb and 6 Mb were also reported and involve LCRA–LCRE and LCRA–LCRG, respectively [8].

According to Portnoï [9], the frequency of 22q11.2 microduplication is approximately half the frequency of microdeletions which can be explained for the highly variable and mild phenotype leading to a low investigation of individuals affected. In addition, the phenotype of individuals with 22q11.2 microduplications can share features with DGS/VCFS, including heart defects, velopharyngeal insufficiency with or without cleft palate or hypernasal speech, and urogenital abnormalities. Here, we described five patients with phenotypic variability that carries deletions or reciprocal duplications at 22q11.2 detected by Chromosomal Microarray Analysis (CMA).

## 2. Materials and Methods

### 2.1. Biological Samples

All five participants had global developmental delay (GDD) without etiological diagnosis after undergoing a thorough clinical evaluation. Assistant physicians from the Goiás state public health system referred each patient to the genetic service at both the Laboratory of Human Cytogenetic and Molecular Genetics and the Biology Department at Pontifical Catholic University in Central Brazil. Parents or guardians signed the informed consent forms approved by the Ethics Committee on Human Research at the Pontifical Catholic University of Goiás (CEPPUC/GO), under the protocol number 1721/2011.

For each proband and their biological parents, a total of 5 mL of peripheral blood was collected using a standard vacuum extraction blood-collecting system containing EDTA and heparin. Genomic DNA was isolated from whole blood using QIAamp DNA Mini kit (Qiagen, Germany), following the manufacturer's instructions. Conventional cell cultures, harvesting, and G-banding at the level of 550 bands were performed in all patients, following standardized procedures [10]. Chromosome observations were performed using a Zeiss Axioscope (Göttingen, Germany) and analyses using the IKAROS software (Metasystems Corporation, Altlussheim, Germany).

### 2.2. Chromosomal Microarray Analysis

Genomic DNA was obtained from peripheral blood from the probands and their parents. The analyses were carried out on the probands and their biological parents to establish whether the DNA rearrangements were* de novo* or inherited. Total DNA (250 ng) for each sample was digested with* NspI,* ligated, PCR amplified and purified, fragmented, biotin-labeled, and hybridized for use in a GeneChip*™* HD CytoScan Array (Affymetrix, Santa Clara, CA, USA). The array was designed specifically for cytogenetic research, including ≈2,696,550 copy number variation markers, 743,304 are single-nucleotide polymorphism markers, and >1,953,246 are nonpolymorphic markers. CEL files obtained by scanning the arrays were analyzed using the Chromosome Analysis Suite software (Affymetrix). Gains and losses that affected a minimum of 50 and 25 markers, respectively, in a 100 kb length were initially considered.

### 2.3. 22q11.2 LCR Structure Analysis

Using the segmental duplication track of the http://genome.ucsc.edu/ browser (Human Genome Build 36.1), we performed an analysis of duplicated genomic sequences including known LCRs (segmental duplication >1 kb of nonrepeat masked sequence with over 90% similarity), comparing the CNV size surrounding the proximal 22q11.2 locus 3 times (chr22: 18,640,000–25,080,000) against itself.

### 2.4. Clinical Report

**Figure 1:**
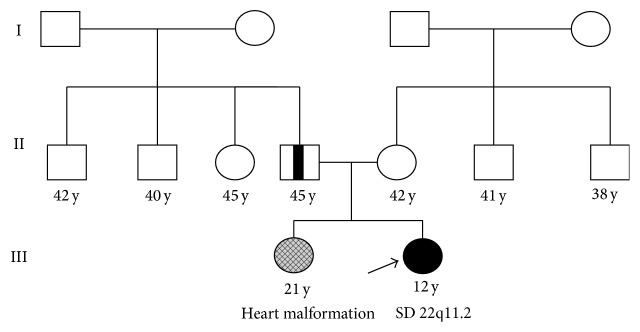
Pedigree of the patient 1 family.

*Patient 1*. Patient 1 was a 12-year-old female patient born to nonconsanguineous parents at 36-week gestation to a 42-year-old mother and 45-year-old father, and her birth weight was 3020 g. Child delivery was carried out through a caesarean section procedure. At 3 months of age, she had epilepsy and reflux. Physical examination of the proband revealed thumb brachydactyly, long finger, retrognathia, short philtrum, and large ear. The family history revealed that her oldest sister has heart malformation (Figure 1). 

**Figure 2:**
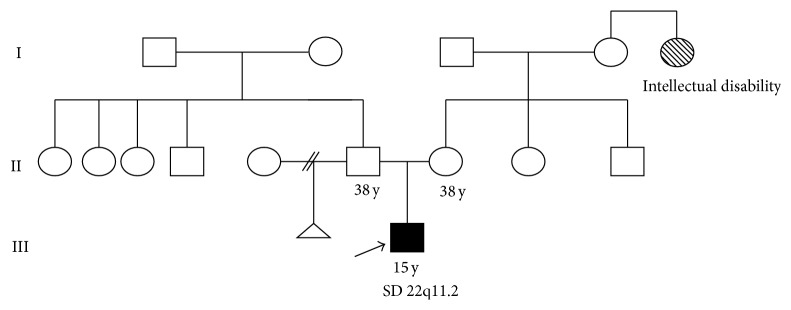
Pedigree of the patient 2 family.

*Patient 2*. Patient 2 was a 15-year-old male patient born to nonconsanguineous parents, at 38-week gestation to a 38-year-old mother and 38-year-old father, and his birth weight was 3200 g and crown-heel length 45 cm. Child delivery was carried out through a caesarean section procedure. After birth, the child showed cyanotic, did not cry, and did not perform the act of sucking. At the age of 14 years, he presented with seizures, cried a lot, had a slow development, and talked nonsense. Physical examination of the proband revealed hypertelorism, ear protuberant with attached lobes, blunted nose, broad nose and narrow bridge, short philtrum, short neck, thumb brachydactyly, clinodactyly of the fifth finger, prognathism, and permanent microdontia. The family history revealed that his aunt's grandmother had intellectual disability (Figure 2). 

**Figure 3:**
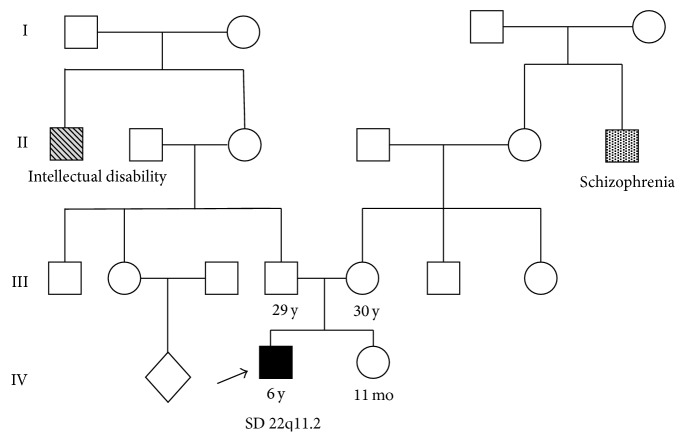
Pedigree of the patient 3 family.

*Patient 3*. Patient 3 was a 7-year-old male patient born to nonconsanguineous parents, at 39-week gestation to a 30-year-old mother and 29-year-old father, and his birth weight was 3840 g and crown-heel length 51 cm. Child delivery was carried out through a caesarean section procedure. Child sat at eight months of age with the physical therapist's help, did not crawl, and had hypotonia. He started walking at one year of age and only spoke at two years of age with the help of a speech therapist. Physical examination of the proband revealed retrognathia, low set ears, ear tags, ear protuberant with attached lobes, low nasal bridge, broad base of the nose, and epicanthal fold. He also had lowered orbital on the right side of the face and ptosis. The family history revealed that a maternal uncle was schizophrenic and paternal uncle had intellectual disability (Figure 3). 

**Figure 4:**
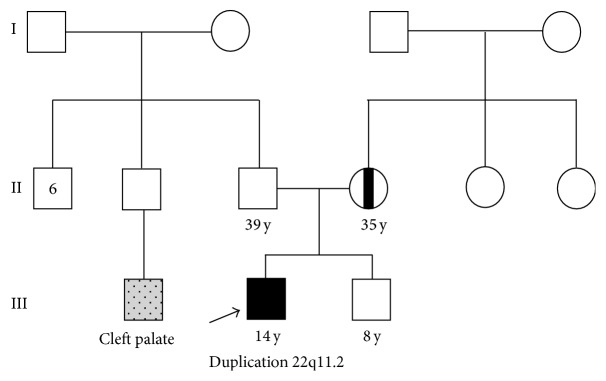
Pedigree of the patient 4 family.

*Patient 4*. Patient 4 was a 14-year-old male patient born to nonconsanguineous parents, at 32-week gestation to a 35-year-old mother and 39-year-old father, and his birth weight was 1600 g and crown-heel length 42 cm. Child delivery was carried out through a caesarean section procedure. Child was born with esophageal atresia and had no suction act and growth delay. His bone age is two years less than the biological age. Physical examination of the proband revealed hypertelorism, micrognathia, thumb brachydactyly, and asymmetric face. The family history revealed that a maternal third cousin was diagnosed with Torre Syndrome and a paternal cousin was born with a cleft lip (Figure 4).

**Figure 5:**
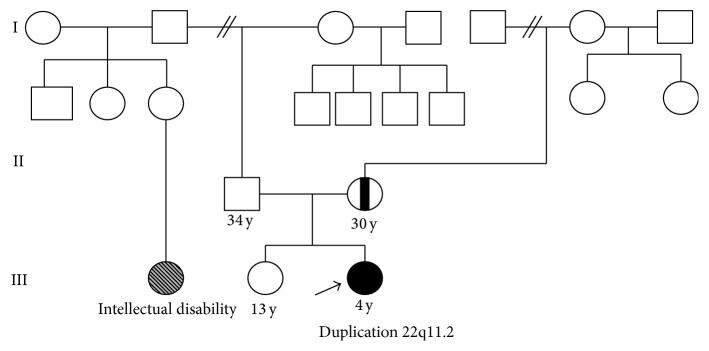
Pedigree of the patient 5 family.

*Patient 5*. Patient 5 was a 4-year-old female patient born to nonconsanguineous parents, at 39-week gestation to a 30-year-old mother and 34-year-old father, and her birth weight was 3140 g and crown-heel length 49 cm. She had jaundice. Child delivery was carried out through a caesarean section procedure. Child sat at eight months and spoke at three years of age. She presents difficulty in walking and has low immunity. Physical examination of the proband revealed hypertelorism, epicanthic fold, low nasal bridge, micrognathia, and thin upper lip. The family history revealed that a father's niece has intellectual disability and difficulty in walking (Figure 5).

## 3. Results and Discussion

In the current study, we describe molecular findings in 5 probands with clinical diagnosis of global developmental delay, presenting microdeletion or microduplication in the apparently identical genomic region within 22q11.2 region and the LCRs which surround these regions. Samples were initially screened using G-banding karyotypes and showed no visible numerical and structural alterations (46,XX or 46,XY). After this analysis, we performed Chromosomal Microarray Analysis that demonstrated abnormalities of 22q11.2 region. All individuals in our study had deletion and duplication breakpoints that flanked or fell within previously characterized LCR22s. For the duplications breakpoints we only identified LCR-H within the 22q11.2 region in contrast with deletions breakpoints that presented LCR22s flanking the 22q11.2 region. The results from CMA and LCRs are shown in Table 1.

**Figure 6:**
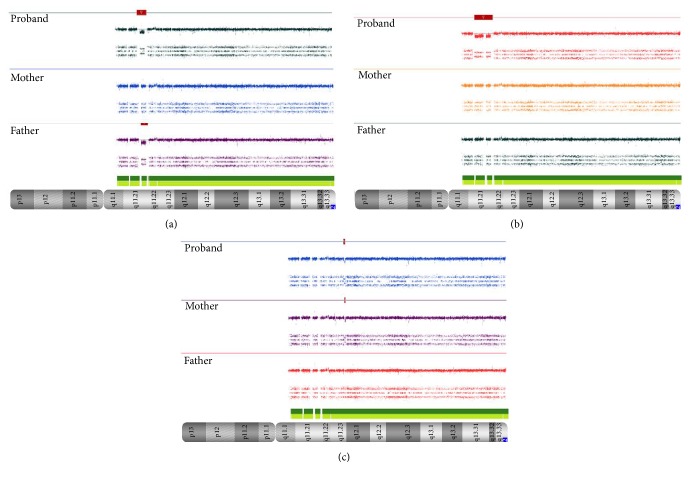
CMA data from probands and their parents at 22q11.2. Analysis showing (a) the bold red line which represents the microdeletion from proband 1 and her parents, (b) 2.88 Mb of* de novo* microdeletion from proband 2 and his parents, and (c) inherited maternal microdeletion from proband 3 and his parents.

We identify three microdeletions at 22q11.2 region from patients 1, 2, and 3 (Figure 6). In patient 2, the CMA showed a* de novo* 22q11.2 microdeletion with 2.88 Mb, flanked by LCR-A and LCR-D, which involves more than 70 genes, including* TBX1* that is suppressed. The majority of individuals have a 3 Mb deletion whose proximal region contains the presumed disease associated with T-box transcription factor 1 (*TBX1)* gene [11–16].* TBX1* gene plays a vital role during development and deletion of this gene leads to a variety of craniofacial and cardiac structures [17]. Rump et al., in 2014, [18] highlighted that loss of* CRKL* combined with loss of either* TBX1* or* MAPK1* in individuals with A–D or larger deletions results in higher rates of cardiac defects than in those individuals with central deletions that involve only* CRKL* gene. Another gene present in this region is the catechol-O-methyltransferase (*COMT*) gene that catalyzes one of the major degradative pathways of the catecholamine transmitters, and one copy of this gene leads to abnormal regulation of catechol-O-methyltransferase levels in the brain. Researchers believe that changes involving this enzyme in the prefrontal cortex may help explain the increased risk of behavioral problems and mental illness associated with 22q11.2 deletion Syndrome [19, 20].

The CMA from patient 1 showed a paternal origin 0.75 Mb deletion at 22q11.2, between LCR-B and LCR-D, classified by Rump et al., 2014 [18] in a Central Group of LCRs. Atypical or distal microdeletions between LCR-B and LCR-D were identified in a limited number of studies [21–26]. Patient 3 demonstrated a* de novo* 0.27 Mb microdeletion at 22q11.2 and we identified only LCRs within this CNV. There are three genes deleted at 0.27 Mb microdeletion:* IGLL3P*,* LRP5L*, and* CRYBB2P.* Haploinsufficiency in seven genes was observed in 0.75 Mb microdeletion (Table 1) that were associated with DGS [15, 27–29]. The* CRKL* gene has also been implicated in the underlying molecular mechanism of 22q11 deletion syndrome. Interestingly, CRKL was mapped in the distal half of the typical deleted region, suggesting that it may be responsible for those cases with the most distal deletions that do not involve* TBX1* gene [30, 31].* TBX1* is the important gene related to the 22q11.2 deletion syndrome and the haploinsufficiency of this gene is presented in most of the phenotype characteristics. However, this gene is not always included in all 22q11.2 deletions, like patients 1 and 3, and many authors suggest that there are regulatory elements or modifier genes mapped far away from this region which may affect the* TBX1* expression [30].

According to Carelle-Calmels et al., 2009, [32] deletion at 22q11.2 was usually sporadic and was reported to be inherited in 6 to 28% of patients with these syndromes. Different from what was observed in patient 1, inherited microdeletions were commonly of maternal origin, and these findings occur due to the recombination rate at 22q11.2 being 1.6 to 1.7 times greater in females than in males [33]. Furthermore, when inherited, these microdeletions had parent-of-origin effect, and phenotypic variability was observed [9].

Patients 1 and 3 presented global developmental delay and ears anomalies; developmental delay or cognitive impairment is reported in over 80% of patients but tends to be relatively mild, with no severe affect and the typical facial gestalt of the proximal microdeletion syndrome was absent. Moreover, ear anomalies are observed in most distal microdeletion and they are nonspecific [11–14].

**Figure 7:**
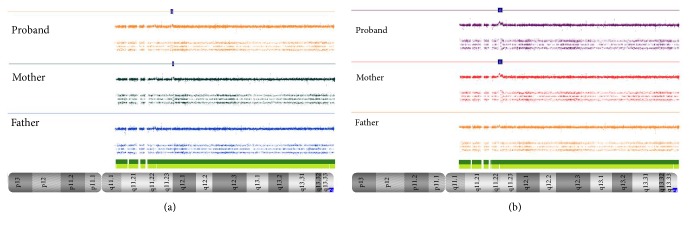
CMA depicts genomic imbalances in chromosome 22. The bold blue line in (a) proband 4 and (b) proband 5 showed the inherited maternal microduplication at 22q11.2.

Additionally, we observed two patients, 4 and 5, who harbor microduplication in 22q11.2 region. Patients 4 and 5 showed maternally inherited duplication of 0.34 Mb and 0.69 Mb, respectively (Figure 7). Both microduplications are located within the block of LCR22-F.* MIR650* gene is located at 0.34 Mb duplication and the potential role of miRNAs in the pathogenesis of duplication phenotypes has to be better investigated. In a study by Merico et al., 2014, [34], the authors showed that the deleted miRNAs in 22q11.2 are involved with the regulation of expression of genes in several developmental pathways which could be affected by miRNAs reduced levels, and the dysregulation of miRNA processing could be due to the haploinsufficiency of* DCGR8* gene [6, 35].

In general, duplications confer milder phenotypes and are more likely to be inherited than their reciprocal deletions, with approximately 93% of small duplications [36]. Although genomic imbalances inherited from a clinically normal parent are usually considered benign CNVs, parents of a child with an inherited chromosome abnormality may sometimes show mild variations of the child's phenotype, such as what have been reported for the 22q11.2 deletion syndrome [2].

Careful clinical assessment of both child and parents is crucial to understand the causative role of duplications at 22q11.2. Considering the unaffected phenotypes of parents, the presence of a genetic modifier has been proposed for other syndromes with variable expressivity. In this context, a genetic modifier is characterized by a combination of CNVs at the same or different loci inherited from parents in whom the single variation was insufficient to cause the disease [37]. Genomic imbalances inherited from phenotypically unaffected parents may contribute to the progeny phenotype through variable penetrance or expressivity, or both, through epigenetic effects, response to environmental factor challenge, modulated epistasis, or stochastic cellular events during fetal development. Moreover, genomic imbalances may uncover a recessive mutation on the nondeleted allele [5, 8, 9].

We need to understand more regarding the reasons for similarities features and widely variable phenotype for individuals with 22q11.2 deletions and duplications. According to Zhang and Lupski, 2015, [38] these variable phenotypes suggested the possible existence of some modifying genetic factors in addition to the large genomic CNVs and recent progress has shown that the SNPs and CNVs in noncoding regions can be the genetic modifiers.

We identified two groups of LCR22 that are flanking the genomic rearrangements from patients 1 and 2. These findings suggest that the occurrence of genomic rearrangements might be mediated by NAHR between the LCRs, increasing the susceptibility to the generation of CNVs. On the order hand, patients 3, 4, and 5 showed LCRs within the reported deletion and duplications breakpoints; thus, it is not possible to speculate the detailed mechanism in relation to how this deletion and duplication initially appeared in these patients. Although high density of long and short interspersed nuclear elements (LINEs and SINEs) around the proximal breakpoint may have been contributory, detailed sequence data characterizing the breakpoints are necessary to recognize these elements and to propose the mechanisms of the genomic rearrangements.

## 4. Conclusions

We observed in our study variable intervals containing known genes and unstudied transcripts as well as the LCRs that are within and often flanking this genomic rearrangement. The recognition of LCRs provides important insights related to the role of genomic architecture in chromosomal rearrangements, chromosome evolution, and human disease. This report further illustrates the potential for determination of small microduplications and microdeletions through of the use of Chromosomal Microarray Analysis. These array-based methods would allow more sensitive and rapid breakpoint localization without the need for multiple FISH experiments. Besides, the applications of microarray allow the identification of distal deletions and also facilitate breakpoint identification in the proximal 22q11.2 deletions and duplications. The identification of these variants is of particular interest because it may provide insight into genes or genomic regions that are crucial for specific phenotypic manifestations and are useful to assist in the quest for understanding the mechanisms subjacent to genomic deletions and duplications.

## Figures and Tables

**Table 1:** Clinical and molecular features of five probands screened by high-resolution CMA technology.

Case	Clinical features^*∗*^	Age (yo)	Sex	CNV	Cytoband	Size (Mb)	Microarray nomenclature	Genes	Origin^*∗∗*^	LCRs start/stop	LCR identity
1	GDD	12	F	Loss	22q11.21	0.75	22q11.21(20,716,902–21,465,662) × 1	ZNF74, SCARF2, MED15, SNAP29, CRKL, LZTR1, SLC7A4	Inherited pat	B–D B–D	99% 99%

2	GDD	15	M	Loss	22q11.21	2.88	22q11.21(18,916,842–21,800,797) × 1	PRODH, DGCR5, DGCR9, DGCR10, DGCR2, DGCR11, DGCR14, GSC2, SLC25A1, CLTCL1, HIRA, MRPL40, UFD1L, CLDN5, SEPT5, GP1BB, TBX1, GNB1L, COMT, ARVCF, DGCR8, DGCR6L, ZNF74, SCARF2, MED15, SNAP29, CRKL, LZTR1, SLC7A4, HIC2	*De novo*	A–D A–D	99% 99%

3	GDD	6	M	Loss	22q11.21	0.27	22q11.23q12.1(25,656,237–25,930,479) × 1	IGLL3P, LRP5L, CRYBB2P1	*De novo*	After H	96%

4	GDD	14	M	Gain	22q11.21	0.34	22q11.23q12.1(25,656,237–25,994,326) × 3	MIR650	Inherited mat	After H	96%

5	GDD	4	F	Gain	22q11.21	0.69	22q11.22q11.23(22,962,196–23,652,512) × 3	POM121L1P, GGTLC2, IGLL5, RTDR1, GNAZ, RAB36, BCR, FBXW4P1	Inherited mat	After H	96%

^*∗*^GDD: Global Developmental Delay; ^*∗∗*^Mat: maternal; Pat: paternal.
